# Modeling of brain tumors using *in vitro*, *in vivo,* and microfluidic models: A review of the current developments

**DOI:** 10.1016/j.heliyon.2024.e31402

**Published:** 2024-05-16

**Authors:** Richu Raju R, Nour M. AlSawaftah, Ghaleb A. Husseini

**Affiliations:** a Biosciences and Bioengineering PhD Program at the American University of Sharjah, Sharjah, United Arab Emirates; b Material Science and Engineering Program at the American University of Sharjah, Sharjah, United Arab Emirates; c Department of Chemical and Biological Engineering, American University of Sharjah, Sharjah, United Arab Emirates

**Keywords:** Brain cancer, Tumor microenvironment, *In vitro*, *In vivo*, Microfluidics

## Abstract

Brain cancers are some of the most complex diseases to treat, despite the numerous advances science has made in cancer chemotherapy and research. One of the key obstacles to identifying potential cures for this disease is the difficulty in emulating the complexity of the brain and the surrounding microenvironment to understand potential therapeutic approaches. This paper discusses some of the most important *in vitro*, *in vivo*, and microfluidic brain tumor models that aim to address these challenges.

## 1 Introduction

The field of brain tumor research is currently at a critical juncture, requiring a thorough analysis of the various methodologies used to address this challenging disease. This thorough review aims to carefully assess the effectiveness and limitations of current brain tumor models, which are essential to the development of novel therapies. Integrating prior research findings and empirical data is crucial in advancing brain tumor research, leading to more effective treatment options and improved patient outcomes in the fight against brain tumors. This review explores laboratory-based (*in vivo* and *in vitro*), animal-based, and cutting-edge microfluidic platforms. Most of the existing reviews only cover the specific models individually, but we aim to consolidate existing studies and findings on brain tumor modeling and present a comprehensive overview that offers researchers and clinicians a holistic perspective on advanced techniques, significant challenges, and emerging directions in this field.

Existing literature showed that in 2019, the number of newly diagnosed brain cancer cases was a staggering figure of 347,922, and 246,253 reported deaths globally [1]. The survival rates for patients diagnosed with brain cancer continue to be disappointingly low, despite notable advancements in treatment modalities. The majority of brain metastases arise from primary neoplasms in the lungs, accounting for approximately 40–50 % of reported cases, followed by breast cancers, which contribute to 15–25 % of instances, and melanomas, which account for 5–20 % of occurrences [2]. Primary brain tumors arise from the neural stem cells (NSCs) or originate from neuronal, astrocytic, and oligodendrocytic lineages. As per the Central Brain Tumor Registry of the United States, Gliomas are the primary brain tumor in the adult population, constituting approximately 30 % of all primary brain and central nervous system (CNS) tumors. High-grade gliomas, exemplified by glioblastomas (GBM) and high-grade astrocytomas (HGA), exhibit a dismal prognosis due to their inherent resistance to conventional radio and chemotherapeutic interventions. In contrast, medulloblastomas (MB) are the prevailing brain cancer type within the pediatric population [3].

Treating brain tumors is quite challenging due to the biological attributes exhibited by these malignancies and the fact that these tumors often grow in parts of the brain that are too hard to reach, even for skilled neurosurgeons and advanced surgical tools, which impedes advancements in therapeutic interventions [4]. Furthermore, the blood-brain barrier (BBB), a network of tight junctions and transport proteins that safeguards the vulnerable neural tissues against potential hazards posed by circulating factors, hampers the accessibility of systemic chemotherapy to the affected regions. Moreover, it is imperative to acknowledge that the distinctive developmental, genetic, epigenetic, and microenvironmental characteristics of the brain often confer strong resistance to both conventional and innovative therapeutic interventions [5].

The brain's complexity emanates from the developmental, genetic, epigenetic, and microenvironmental factors that affect it. Neurogenesis and synaptogenesis create the brain's neural architecture, facilitating the establishment of neural circuits. Genetically, the brain's detailed structure is crucial for controlling genes that affect its structure and functionality. This includes neurotransmitter receptors, which are of utmost significance in facilitating the process involved in synaptic transmission [6]. Epigenetic mechanisms, including but not limited to DNA methylation and microRNA regulation, serve as pivotal regulators of gene expression by exerting their influence without causing any modifications to the underlying genetic code. The microenvironment, which encompasses the network of the extracellular matrix, the complex interplay of neurotrophic factors, and the dynamic regulation of cerebral blood flow, assumes a paramount role in providing essential support for the processes of neural development and the delicate maintenance of optimal brain health. The multifaceted interplay of these various factors serves as a crucial determinant in shaping the brain's remarkable ability to withstand challenges while also rendering it vulnerable to different disorders. This underscores the significance of acquiring a comprehensive understanding of these mechanisms, as it holds the key to propelling advancements in neuroscience and facilitating the development of potential therapeutic interventions [7].

**Fig. 1:**
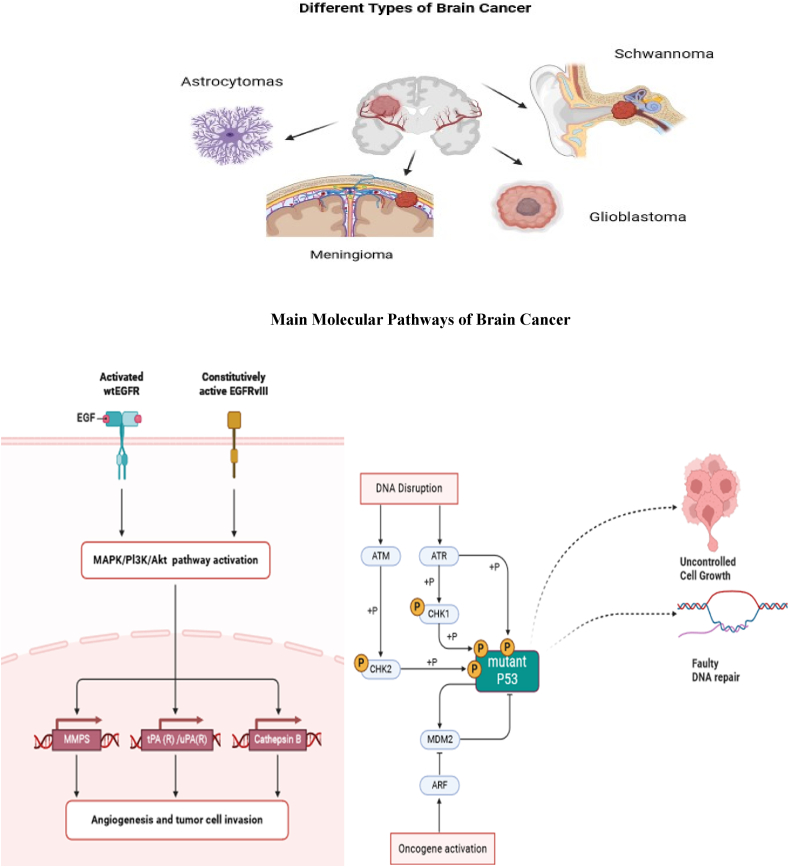
Overview of various brain tumors(created using BioRender.com).

Due to the complex nature of the brain tumor ecosystem, it is very important to create models that accurately show how brain tumors start and metastasize. This will help scientists develop better ways to treat brain tumors and better drugs. *In vitro* tumor models offer valuable insights into the molecular and cellular mechanisms underlying the pathological progression of tumor vasculature, particularly in relation to the development of brain tumors. *Ex vivo* tumor models aid in the study of cancerous tissues excised from a living organism in a controlled laboratory environment. These models utilize brain tumor cell cultures, organoids, and tissue slices [8]. They synergistically augment *in vivo* and clinical investigations, advancing brain tumor treatment. The utilization of *in vivo* models for brain tumors, encompassing xenografts, genetically engineered mouse models (GEMMs), patient-derived xenografts (PDX), and various other modalities, represents indispensable methodologies in brain tumor research. The models above capture the complex dynamics of tumor growth in living organisms, primarily in rodents. The selection of an appropriate model by researchers is contingent upon their research objectives, thereby enabling them to acquire profound insights into the process of tumor development and effectively assess the viability of therapeutic approaches prior to embarking upon clinical trials [9]. This paper will examine the various models available in the literature to mimic or replicate the characteristics and features of brain tumors. Fig. 1 below briefly overviews the common models used in research to study brain tumors.

The following section will give a brief overview of the types of common brain tumors.

## 2 Types of commonly diagnosed brain tumors

Studies have reported approximately 130 different types of brain tumors, ranging from the most common to relatively rare tumors. Some of the most commonly diagnosed brain tumors include gliomas, medulloblastomas, meningiomas, astrocytomas, and schwannomas.

### 2.1 Gliomas

Gliomas originate from the glial cells, such as astrocytes, oligodendrocytes, and ependymal cells. Astrocytomas, oligodendrogliomas, and ependymomas represent distinct subtypes of gliomas, a heterogeneous group of primary brain tumors [10]. These gliomas are classified according to the World Health Organization (WHO) grading system into three grades: Grade II, denoting low-grade tumors, Grade III, indicating anaplastic tumors; and Grade IV, representing the most aggressive form known as GBM. GBM, which accounts for the majority of cases (at a rate of 65 %), represents the most prevalent histological subtype with the highest degree of malignancy [11]. They have conventionally been classified into two primary classifications: 'diffuse' gliomas and 'non-diffuse' gliomas. Diffuse gliomas exhibit a distinctive feature wherein tumor cells have the ability to migrate extensively within the CNS parenchyma, rendering surgical resection unattainable.GBM's histological characteristics encompass prominent hypercellularity, nuclear atypia, microvascular proliferation, and necrosis. The observed neoplastic growth exhibits a distinctive arrangement of tumor cells forming palisades surrounding areas of necrosis [12]. The accurate grading and classification of various gliomas is contingent on their histological attributes and on the molecular characteristics encompassing altered genes. Molecular markers play a pivotal role in elucidating and managing gliomas, thereby furnishing indispensable insights into their genetic and molecular profiles/pathways. Some of the most prominent indicators are Isocitrate Dehydrogenase (IDH) mutations, which are suggestive of a more favorable prognosis in lower-grade gliomas, and MGMT (O6-methylguanine-DNA methyltransferase) promoter methylation, which is a predictive factor for enhanced responses to specific chemotherapeutic interventions [12,13]. The co-deletion of the short and long arms of 1p and 19q suggests a distinctive genetic alteration observed in oligodendrogliomas. To add on, Epidermal Growth Factor Receptors (EGFR) amplification and mutations on the tumor cells, TP53 alterations, and ATRX mutations have also been seen in the pathological examinations of several gliomas [14]. Moreover, it is worth noting that H3K27 M mutations were linked to unfavorable prognoses in certain pediatric gliomas. In addition, the presence of Telomerase Reverse Transcriptase (TERT) promoter mutations and Platelet-Derived Growth Factor Receptor Alpha (PDGFRA) markers offer valuable additional information regarding the behavior of gliomas and their potential for effective treatment strategies [15]. Utilizing these markers facilitates the customization of therapeutic approaches for individual patients, thereby enhancing the overall management and provision of care for individuals afflicted with glioma.

### 2.2 Medulloblastomas

MB is the predominant CNS embryonal cancer. Recent investigations have successfully delineated four discrete molecular subgroups, namely WNT, Sonic Hedgehog (SHH), Group 3, and Group 4, which exhibit distinctive genetic and molecular attributes. The WNT subgroup exhibits robust activation of the highly conserved WNT signaling pathway, a critical regulator of cellular processes such as proliferation and differentiation that play pivotal roles in the orchestration during early embryonic growth [16]. In stark contrast, the SHH subgroup manifests dysregulated Sonic Hedgehog signaling, which plays a pivotal role in tissue patterning during the nascent stages of nervous system development. They infiltrate the adjacent cerebral parenchyma and disseminate to various regions within the CNS. MBs tend to manifest treatment resistance, a phenomenon linked to many genetic and molecular determinants [17].

### 2.3 Meningiomas

Meningiomas are the prevailing primary tumors affecting the brain, often originating from the meninges, which are the protective membranes enveloping the brain and spinal cord. The majority of these tumors have a benign nature (Grade I) and demonstrate a modest growth rate. However, a small proportion may exhibit atypical characteristics (Grade II) or malignancy (Grade III), indicating an increased likelihood of recurrence and aggressive behavior. The symptoms of the tumor vary depending on its location and size, including headaches, visual impairments, and more severe neurological impairments. Diagnosis is often accomplished using imaging modalities such as MRI or CT scans, while treatment choices include meticulous surveillance, surgical excision, radiation therapy, or pharmacotherapy, contingent upon the tumor's attributes and the patient's well-being. The prognosis for persons diagnosed with benign meningiomas is often positive, particularly when the tumor can be entirely excised. However, those with atypical or malignant types of meningiomas have a more difficult outlook owing to the potential for recurrence and infiltration into adjacent organs [18].

### 2.4 Astrocytomas

Astrocytomas are tumors originating from astrocytes, a subset of glial cells found in the CNS. These entities are categorized according to their histological attributes and molecular properties. The diagnosis, prognosis, and therapy of astrocytomas have been influenced by the identification of several subtypes and indicators via molecular research. Research has shown many molecular subcategories of high-grade astrocytomas, including the proneural, proliferative, and mesenchymal subcategories. Each subcategory is linked to specific molecular characteristics and clinical results. Moreover, the deactivation of the neurofibromin 1 (NF1) gene has been associated with heightened proliferation of glial progenitor cells and the emergence of optic gliomas, providing insights into the genetic pathways that underlie the development of astrocytomas [19]. The WHO classified them into four grades based on their aggressiveness, ranging from Grade I, which is the least aggressive and often considered benign, to Grade IV astrocytomas, which are highly malignant and aggressive.

### 2.5 Schwannomas

Schwannomas, also referred to as neurilemmomas, are non-malignant neoplasms originating from Schwann cells, responsible for synthesizing the myelin sheath enveloping peripheral nerves. Typically, these neoplasms have a sluggish growth rate and lack malignancy, often originating in the cranial nerves, spinal nerve roots, and peripheral nerves. Vestibular schwannoma, also known as acoustic neuroma, is the prevailing form of schwannoma. It specifically targets the nerve that governs balance and hearing, resulting in symptoms such as hearing impairment, tinnitus, and imbalance. Schwannomas have the capacity to exert compression on neighboring tissues during their growth, which may result in symptoms such as pain, numbness, weakness, or impaired functionality within the afflicted region [20].

### 2.6 Ependymal tumors

Ependymal tumors are rare cancers that affect the CNS and start from ependymal cells. The ependymal cells, which are situated along the ventricles of the brain as well as the central canal of the spinal cord, fulfill a crucial function in the generation and propagation of cerebrospinal fluid [21]. The occurrence of these tumors can manifest in both pediatric and adult populations, thereby necessitating their classification into diverse subtypes predicated upon their anatomical site, histological grade, and distinctive attributes. Several molecular markers are significantly expressed in ependymal tumors [22]. Firstly, studies have shown that these tumors have the occurrence of v-relavian reticuloendotheliosis viral oncogene homolog A (RELA) fusion, a genetic modification characterized by the involvement of the RELA gene, which is a commonly observed phenomenon in supratentorial ependymomas. The presence of the YAP1-MAMLD1 fusion gene has been observed in a subset of pediatric posterior fossa ependymomas. Furthermore, the identification of additional markers such as TP53 mutations, loss of chromosome 22q, C11, or f95-RELA fusion, and c-Myc amplification offers significant insights into the tumor's characteristics, progression, and potential therapeutic targets [23].

## 3 A brief history of brain tumor modelling

The development of brain tumor modeling is a fundamental aspect of the progress of neuro-oncology, and it is characterized by notable historical achievements that have laid the foundation for current research approaches. The introduction of early animal models, including the creation of the first mouse models in the early 20th century, represented a significant change in our capacity to investigate brain tumors in a sophisticated creature that closely resembles the pathophysiology of human diseases. The utilization of animal models has played a pivotal role in the advancement of knowledge about tumor biology, the microenvironment of the brain, and the systemic impacts of brain tumors [24].

Historical advancements in brain tumor modeling have played a vital role in influencing the current state of brain tumor research. A notable achievement was the creation of the first animal models for brain cancers. The use of animal models facilitated the examination of tumor formation, progression, and therapy response inside a controlled setting by researchers. The first recorded use of animal models for brain tumor investigation may be traced back to the early 20th century when experts like Cushing and Bailey performed investigations in the 1920s. The use of these first animal models established the foundation for further progress in comprehending the biological aspects of brain tumors.

The 1970s saw a significant advancement in brain tumor research with the introduction of the first *in vitro* tumor models. Scientists successfully cultivated tumor cells in a controlled laboratory environment, allowing them to investigate the behavior of tumor cells. This advancement brought about a significant transformation in the realm of cancer research and established a foundation for evaluating possible treatments and comprehending the molecular pathways that drive tumor proliferation. It is assumed that the inception of the first *in vitro* tumor models took place around the mid-1970s [25].

The identification of cancer stem cells inside human brain tumors represents an essential breakthrough in the field of brain tumor modeling. In the early 2000s, around 2003, the first *in vitro* discovery and characterization of cancer stem cells derived from human brain tumors was documented. The aforementioned finding provided valuable knowledge on the hierarchical structure of tumors and the involvement of cancer stem cells in the beginning and advancement of cancers.

The revolutionary milestone of the comprehensive genomic study of human glioblastoma multiforme in 2008 provided valuable insights into the genetic composition of brain malignancies. The work revealed significant genetic modifications, including isocitrate dehydrogenase (IDH1) mutations, which play a crucial role in glioblastoma development. The comprehension of the genetic underpinnings of brain tumors has played a pivotal role in the development of focused therapeutic interventions and individualized treatment strategies. Brain tumor research has been profoundly influenced by the progress made in molecular diagnostics. The advent of molecular diagnosis of brain cancers has been facilitated by recent advancements in brain tumor-related gene research and genomic testing technology. Notably, this field has made substantial progress during the last decade. These technological breakthroughs have facilitated more accurate categorization of brain tumors and have facilitated the development of individualized treatment approaches that rely on the molecular characteristics of each tumor.

The use of microfluidic models has dramatically enhanced the investigation of brain tumors by offering a precise platform to replicate the tumor microenvironment. The inception of microfluidic models for brain tumors can be traced to the early 21^st^ century, during which researchers initiated the development of advanced *in vitro* systems for investigating tumor activity and treatment reactions.

The significance of microfluidic 3D cancer models has grown in recent years due to their growing relevance in fundamental cancer biology, medication screening, and drug development endeavors. Researchers have achieved significant breakthroughs in developing and assessing microfluidic *in vitro* models of the blood-brain barrier. These developments include the use of chip materials, porous membranes, endothelial cells, shear stress, and tight junction indicators. Current advancements have emphasized the use of microfluidic devices for investigating metastatic mechanisms, including cellular infiltration, intravasation, extravasation, and tumor formation of blood vessels. These systems provide a dynamic platform for investigating the complex interactions between tumor cells and the microenvironment, providing vital insights into the evolution of tumors and their response to therapeutic interventions. The use of microfluidic models has greatly enhanced the investigation of brain tumors by offering a precise platform to replicate the tumor microenvironment. The inception of microfluidic models for brain tumors can be traced to the early 21^st^ century, during which researchers initiated the development of advanced *in vitro* systems for investigating tumor activity and treatment reactions.

The significance of microfluidic 3D cancer models has grown in recent years due to their growing relevance in fundamental cancer biology, medication screening, and drug development endeavors. Researchers have achieved significant breakthroughs in developing and assessing microfluidic *in vitro* models of the blood-brain barrier. These developments include the use of chip materials, porous membranes, endothelial cells, shear stress, and tight junction indicators.

Current advancements have emphasized the use of microfluidic devices for investigating metastatic mechanisms, including cellular infiltration, intravasation, extravasation, and tumor formation of blood vessels. These systems provide a dynamic platform for investigating the complex interactions between tumor cells and the microenvironment, providing vital insights into the evolution of tumors and their response to therapeutic interventions.

## 4 Molecular classifications of brain tumors

The development of molecular classification systems for brain cancers is a very influential change in neuro-oncology, enhancing our comprehension of tumor biology and greatly affecting the methods used for diagnosis, prognosis, and therapy. The grading systems developed by the WHO have been crucial in this development since they integrate molecular markers with histological characteristics to provide a more precise classification of brain tumors. Historically, the categorization and evaluation of brain tumors were based on the microscopic analysis of tumor tissue, which included analyzing cellular structure, mitotic function, cell death, and blood vessel growth [26]. WHO grading system, first implemented in the 1970s and subsequently revised, classifies brain tumors into Grade I (indicating the least aggressive) to Grade IV (indicating the most aggressive) according to certain characteristics. Although this technique played a crucial role in establishing a uniform diagnosis and providing prognostic data, it had drawbacks stemming from its subjective character and the potential for varying interpretations.

In the late 20^th^ and early 21^st^ centuries, molecular biology tools emerged, enabling the investigation of the genetic changes that underlie brain tumors. One of the first advancements was the detection of 1p/19q codeletion in oligodendrogliomas, as well as the finding of mutations in the IDH1 and IDH2 genes in gliomas. The results of this study demonstrate that cancers exhibiting comparable histological characteristics may possess distinct genetic profiles, leading to varying clinical manifestations and therapeutic responses [27].

The integration of genetic data with histology was a significant development in the 2016 revision of the WHO categorization of CNS cancers. For example, the classification of gliomas was revised by considering their IDH mutation status and the presence or absence of 1p/19q codeletion. This resulted in establishing more specific diagnostic categories, namely "astrocytoma, IDH-mutant" and "oligodendroglioma, IDH-mutant and 1p/19q-codeleted." The molecular stratification yielded more precise prognostic data and facilitated the customization of therapy strategies [28].

## *5* *In vivo* models of brain tumors

**Fig. 2:**
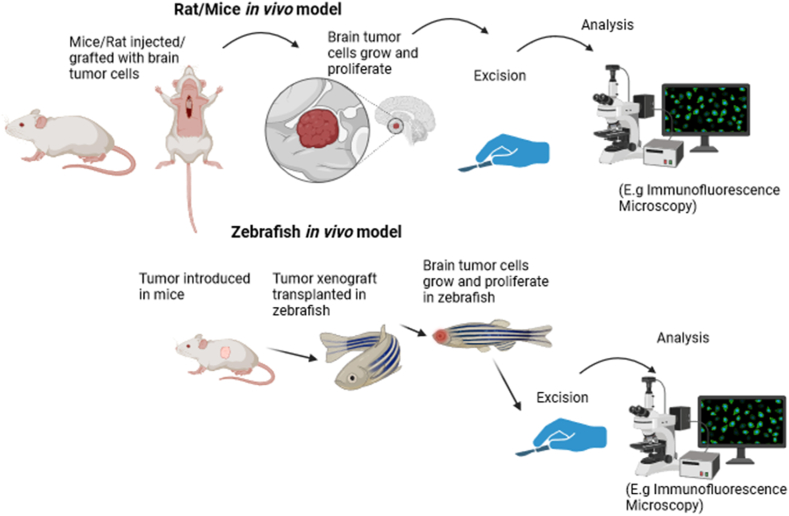
A generalized overview of the workings of an *in vivo* brain tumor model.

Over the course of the last six decades, a plethora of animal models have been carefully designed and refined with the primary objective of comprehensively investigating the processes underlying the initiation and progression of brain tumors. The categorization of models can be divided into three distinct categories: chemically induced models, GEM models, encompassing virally induced models, and xenograft models. While it is undeniable that these models have made substantial strides in elucidating the mechanisms underlying tumor initiation and progression, the translation of this knowledge into more efficacious treatment modalities has been somewhat constrained [8]. This can be attributed to a multitude of factors. Primarily, the dissimilarities between the tumor models employed *in vitro* and the biological characteristics of patient tumors contribute significantly to this discrepancy. Additionally, the pharmacokinetic profiles exhibited by the animal subjects differ from those of humans, further influencing the efficacy of treatment strategies. Moreover, the tumors established in animal models fail to accurately represent the cellular heterogeneity observed in human tumors [29]. Fig. 2 briefly expresses the basic concept behind the *in vivo* model of brain tumors.

Historically, the utilization of chemically induced brain tumors has been employed as a valuable approach to investigate the nature of gliomas and various other tumor subtypes. The predominant techniques employed for the induction of brain tumor formation encompass the utilization of N-nitrosourea and carcinogenic viruses, namely RSV-1 and human adenovirus. The utilization of cell lines derived from murine and rodent brain tumors has been extensively employed in scientific research. Notable examples encompass RG2, BT7C, CNS1, C6, and 9L [30]. A study by Hormuth et al. used a chemically induced C6 glioma model to investigate the therapeutic effects of whole-brain radiation therapy in a cohort of 12 rats [31]. A potential application of these chemically induced models lies in their capacity to facilitate the inference of knowledge pertaining to chemically induced mutagenesis within the CNS. Nevertheless, it is important to note that while numerous compounds have demonstrated their ability to induce tumorigenesis in the nervous system of rats, the identification of a specific chemical agent responsible for the development of brain tumors in humans remains elusive. The observed incongruity likely pertains to the circumstance that in carcinogenesis trials, rodents have intentionally been subjected to pharmacologically toxic dosages via a solitary bolus injection during an early developmental phase or through repeated administration over several months during adulthood. In stark contrast, it is highly probable that in the context of human beings, exposure predominantly transpires in an intermittent manner, characterized by the presence of minute quantities. In the context of rat carcinogenesis experiments, it has been observed that the susceptibility of developing embryos to tumor development surpasses that of pregnant females by a factor ranging from 50 to 100. Hence, owing to variations in chemical dosages, durations of exposure, metabolite affinities, and kinetics across different species, the utilization of chemically induced models has yielded only a restricted degree of understanding regarding the causation of central nervous system neoplasms in humans.

The utilization of GEM models has undeniably advanced the field of brain tumor research by allowing researchers to meticulously manipulate the genetic composition of these models, thereby faithfully mimicking the crucial mutations observed in human brain tumors. In the realm of GBM investigation, it is preferred to employ GEM models that encompass the upregulation of platelet-derived growth factor (PDGF) or the expression of the mutated variant of the epidermal growth factor receptor (EGFRvIII), thereby emulating genetic occurrences linked to the progression of glioblastoma [32]. In the context of MBs, it is noteworthy to mention that GEM models are employed to manipulate the SHH pathway. This manipulation is undertaken to replicate the disruptions associated with the formation of MBs, particularly in cases involving pediatric patients. Incorporating mutations in the IDH1 gene within oligodendroglioma models represents the genetic milieu observed in human oligodendrogliomas [33]. The primary emphasis of Meningioma GEM models revolves around investigating mutations occurring in the Neurofibromatosis 2 (NF2) gene. Conversely, ependymoma models primarily explore genetic modifications, such as the occurrence of REL-A fusions. The GEM models employed in this study serve as valuable tools for examining highly regulated systems that govern the complex molecular mechanisms implicated in the pathogenesis of brain tumors. By utilizing these models, researchers can better understand the processes involved in tumor initiation and progression, and identify potential therapeutic targets. By employing these computational models, scholars are able to undertake preclinical investigations, thereby expanding our understanding of cerebral neoplasms and expediting the formulation of precise and efficacious therapeutic approaches.

Both rat and mouse glioma models are commonly utilized in scientific investigations to examine gliomas, a form of brain tumor originating from glial cells. Rat models are employed due to their larger size, which enables the execution of surgical procedures and facilitates the monitoring of tumor growth. Conversely, mouse models are preferred for their cost-effectiveness and frequent utilization of genetic modifications to replicate human glioma-associated mutations. The induction methods for glioma exhibit considerable variation, with mouse models predominantly employing genetic modification techniques, while chemical induction can be employed in both mouse and human species. The dissimilarities in tumor characteristics and molecular attributes between the two models are frequently taken into account by researchers when making a selection, based on their specific research objectives and the accessibility of pertinent tools and reagents. Researchers may opt to synergistically integrate both computational models in order to acquire a holistic comprehension of the dynamics underlying glioma biology and the prospective therapeutic interventions. The optimal glioma model should exhibit consistency with human GBM in relation to its morphological attributes, metastatic capacity, vascular dynamics, and immune microenvironment. A myriad of cell lines that faithfully recapitulate the complex characteristics of human GBM have been extensively employed in the realm of scientific investigation, namely U251 and U87, as well as murine cell line GL261, and rat cell lines 9L/LacZ, F98, RG2, CNS-1, and C6. The xenograft models U251 and U87 are exclusively amenable to development within immunocompromised rodent hosts, in contrast to the remaining cell lines, which can be cultivated in immunocompetent syngeneic models.

The C6 glioma model represents a widely employed experimental paradigm within the realm of glioma research. The experimental procedure entails the intracranial introduction of C6 glioma cells, initially obtained from a chemically induced rat glioma, into the rodent's cerebral cortex. Tumorigenic formations derived from C6 glioma cells manifest robust attributes akin to those observed in human glioblastomas, thereby rendering this experimental model amenable for investigating the pathophysiology of rapidly proliferating and infiltrative cerebral neoplasms. Researchers have widely employed the utilization of the C6 glioma model to explore various aspects of glioma biology, encompassing tumor growth, invasion, angiogenesis, and the evaluation of therapeutic interventions. Although this particular model presents the inherent benefit of providing a reliable depiction of tumor behavior and leveraging extensively validated data, it does, however, possess certain limitations in its ability to faithfully reproduce the genetic intricacies observed in human glioblastomas. However, it is important to acknowledge that the C6 glioma model continues to serve as a valuable instrument in pursuing enhanced comprehension and therapeutic interventions for these profoundly malignant cerebral neoplasms [32,34].

### 5.1 Rat brain tumor models

A study by Liu et al. used spectral computed tomography to evaluate rat C6 glioma. To ensure the reliability and validity of the findings, 10-week-old male Wistar rats were procured and subsequently accommodated within a meticulously maintained specific pathogen-free facility, thereby minimizing the potential for any confounding variables that may arise from external sources. The experimental cohort consisted of anesthetized rodents immobilized on a stereotactic apparatus, followed by the administration of a surgical intervention involving the intracranial injection of C6 glioma cells. The monitoring of tumor growth was conducted, and CT scanning was carried out on day 12 following injection in response to the accelerated proliferation of C6 gliomas. Notably, the aforementioned gliomas have been observed to induce rat mortality within a 3-4-week time frame in the absence of any form of intervention [35].

Xenograft models, indispensable in preclinical investigations, encompass human brain tumor cells or tissue transplantation into immunodeficient mice. This particular methodology facilitates the investigation of tumor proliferation dynamics and the subsequent evaluation of therapeutic interventions within an *in vivo* model. PDXs leverage tumor tissue procured directly from patients, thereby preserving the genetic and histological attributes of the primary tumor, thus affording a more clinically pertinent depiction. The subcutaneous and orthotopic implantation methods can be employed for tumor establishment, with the latter offering a superior physiological microenvironment, albeit accompanied by technical intricacies. Researchers meticulously monitor tumor growth and treatment responses using advanced imaging modalities such as MRI or bioluminescence imaging. Although xenograft models may not exhibit the immune system interactions observed in syngeneic models, their simplicity, cost-effectiveness, and scalability advantages render them highly valuable for initial drug screening and therapeutic advancements in brain tumor research. A study by Joo et al. developed patient-specific orthotopic xenograft models to understand the biology of human GBM in situ. Orthotopic models were established by utilizing primarily cultured GBM cells. A precise stereotactic injection technique introduced acutely dissociated GBM cells into the brains of immunodeficient NOG mice. The injection coordinates were carefully determined as 2 mm to the left and 1 mm anterior to the bregma, with a depth of 2 mm from the dura. This injection procedure was performed within a 12-h timeframe following the surgical intervention. Mice exhibiting a substantial decrease in overall body weight exceeding 20 % were subjected to euthanasia, following which their brains were subjected to either paraffin or frozen section processing methodologies. The models effectively recapitulated the pathological and genomic characteristics exhibited by the parental tumors. The treatment response exhibited by the parental tumors was successfully replicated in the animal models, thereby validating the translational potential of these models. Furthermore, through meticulous analysis, the team could identify distinct molecular signatures directly associated with the clinical aggressiveness of GBM [36].

Another study by Wu et al. investigated the effects of sonodynamic therapy on tumor reduction in a C6 rat glioma model. The present study involved executing experiments utilizing a C6 intracranial glioma tumor model in a cohort of 37 male Sprague Dawley rats. The interventions were executed approximately seven days after the implantation of the tumor, at which point the tumor attained a diameter ranging from 1 to 3 mm, ascertained through the utilization of MRI. A dosage of 60 mg/kg of 5-aminolevulinic acid (5-ALA) was administered via injection, precisely 6 h prior to the sonication procedure. The application of MR-guided focused ultrasound (MRgFUS) at a frequency of 1.06 MHz was administered continuously, with an in situ spatial-peak temporal-average intensity of 5.5 W/cm^2^, over 20 min. The acquisition of MR thermometry was undertaken to meticulously observe and analyze the fluctuations in temperature within the cerebral region during the sonication process. The assessment of tumor growth response in the experimental cohorts, namely those administered with 5-ALA in isolation, focused ultrasound (FUS) in isolation, the combination of 5-ALA and FUS, as well as the control group, was conducted through the utilization of MRI on a weekly basis after the respective treatments. Magnetic Resonance-guided Focused Ultrasound (MRgFUS) was applied at an intensity of 5.5 Watts per square centimeter for 20 min, causing increases in temperature, within the brain tumor, from an initial range of 32.3 ± 0.5 °C to a subsequent range of 33.2 ± 0.9 °C. Similarly, the temperature range increased from 37.2 ± 0.7 °C to 38.4 ± 1.1 °C. The group that received 5-ALA combined with FUS demonstrated a notable enhancement in tumor growth suppression and overall survival. This effect was observed when the core body temperature was initiated at either 32 °C or 37 °C. The administration of 5-ALA in isolation, as well as the application of FUS as a standalone intervention, did not yield any significant enhancements in terms of survival outcomes. The findings of this study suggest that the utilization of low-power continuous wave transcranial MRgFUS, in combination with 5-ALA, exhibits a suppressive impact on the growth of brain tumors in rats, even in the absence of a thermal dose [37].

### 5.2 Mice brain tumor models

Taillander et al. conducted a study to investigate the most effective therapeutic modalities for the treatment of human malignant brain tumors *in vivo* using two distinct models. These models involved tumor cells' heterotopic and orthotopic xenografting into nude mice. In the initial phase of implantation, a total of 11 high-grade gliomas and 4 low-grade tumors were meticulously grafted onto the epigastric vessels of human subjects. 11 high-grade gliomas, devoid of any low-grade tumors, were successfully engrafted into immunodeficient nude mice. Subsequently, it was observed that the aforementioned mouse-adapted gliomas successfully engrafted into additional immunodeficient murine hosts, thereby manifesting progressive growth and development. When transplanted onto the murine brain, the gliomas of human origin exhibited a remarkable capacity to infiltrate the host brain at considerable distances from the primary tumor, akin to the observed phenomenon in human patients. Utilizing a second implantation technique served as a pertinent model for comprehending the progression of human gliomas. This model enabled a comprehensive investigation of the migratory behavior of malignant cells within the cerebral region, both before and after therapeutic interventions, ascertained through the heterotopic model. Furthermore, it facilitates an evaluation of the brain's capacity to withstand these chemotherapy and radiotherapy [38]. Mendez et al. successfully generated a novel endogenous mouse model of mACVR1 brainstem glioma, thereby contributing to the expanding body of knowledge in the field of glioma research. Histological analysis was employed to characterize and validate the model in question effectively. RNA-sequencing analysis was conducted on neurospheres containing mACVR1. The study involved the transplantation of mACVR1 neurospheres into the pons region of mice with intact immune systems. The aim was to evaluate the therapeutic effectiveness and potential adverse effects of immune-stimulatory gene therapy utilizing adenoviruses that express thymidine kinase (TK) and fms-like tyrosine kinase 3 ligand (Flt3L). Neurospheres expressing the surrogate tumor antigen ovalbumin were generated using mACVR1 in order to investigate the potential induction of tumor antigen-specific T-cells recruitment through TK/Flt3L treatment. The histological examination of mACVR1 tumors reveals their specific localization within the brainstem, accompanied by a notable augmentation in the downstream activation of the bone morphogenetic pathway. This enhanced signaling is substantiated by the observed elevation in levels of phosphor-smad1/5 and Id1. The transcriptomic analysis conducted on mACVR1 neurospheres revealed a notable upregulation in the TGFβ signaling pathway, accompanied by the modulation of cellular differentiation processes. The administration of TK/Flt3L via adenoviral vectors in murine models harboring brainstem gliomas led to the induction of potent antitumor immune responses, characterized by the infiltration of tumor-specific T lymphocytes and a notable extension in the median survival of the experimental subjects.

It has been observed that invasive, intracranial tumors can be successfully established by utilizing a heterotopic-to-orthotopic approach. This particular methodology involves the initial establishment of tumors through direct transplantation of human biopsies into the flanks of immunodeficient mice. The serial passaging of xenografts in animal models is a straightforward process. It is of great interest to note that upon the establishment of the tumors as intracranial tumors, a remarkable resemblance to human tumors was observed, characterized by the presence of highly invasive tumors that frequently exhibited EGF receptor amplification. Furthermore, it is worth noting that the DNA copy number and mRNA expression profiles of a diverse array of subcutaneous tumors exhibited remarkable resemblances to those observed in human tumors. Furthermore, the tumors under investigation demonstrated a notable dearth in endothelial cell proliferation, thereby shedding light on an intriguing aspect of their pathophysiology. The observed phenomenon of limited angiogenesis in the mouse brain may be attributed to the comparatively diminutive size of the tumor.

### 5.3 Zebrafish brain tumor models

Zebrafish, as an alternative experimental *in vivo* model, possess the capacity to manifest tumors that exhibit histological and genetic resemblances to their human counterparts, thereby rendering them a valuable resource for the study of cancer in humans. Zebrafish models exhibit remarkable amenability to high-throughput screening techniques, thereby rendering them highly suitable for drug discovery. Additionally, these models have demonstrated their potential for the transplantation of primary patient tumors, further enhancing their utility in the field of biomedical research [39]. Zebrafish emerge as a highly advantageous and efficient substitute for conventional *in vivo* tumor models, including rodents, due to their remarkable cost-effectiveness and time efficiency. In the past few years, a multitude of pediatric brain tumor models have been successfully established in the zebrafish, serving as valuable tools for elucidating the molecular pathways underlying tumorigenesis. This encompasses the comprehensive examination of distinct and overlapping molecular cascades underpinning pediatric high-grade gliomas (HGG), both within the confines of the brainstem and beyond, to discern three distinct molecular subcategories of diffuse intrinsic pontine glioma (DIPG) [40]. In a study undertaken by Mayhrofer et al., a zebrafish model of brain tumors was meticulously crafted. This model was established through the somatic expression of oncogenes, specifically those that activate MAPK and PI3K signaling pathways in neural progenitor cells. The researchers astutely observed that HRASV12, among the various oncogenes investigated, exhibited the highest efficacy in inducing both heterotopia and invasive tumors. In order to generate a model of brain tumor, the researchers employed the Gal4-UAS system, a widely utilized tool in genetic studies. This system enables the induction of expression of various oncogenes under the UAS promoter, which is present in the driver line Et(zic4:GAL4TA4, UAS:mCherry)hmz5. In stark contrast to extant rodent models pertaining to brain dysplasia or brain tumors, the zebrafish glioma model offers a distinct advantage by facilitating the concurrent emergence of tumors and heterotopia in a nearly equivalent proportion. This occurrence is induced by the same oncogene, thereby allowing the scrutiny of the mechanisms governing fate determination and the prerequisites for progression. Furthermore, the proposed model facilitates the meticulous examination of temporal dynamics associated with tumor advancement within a living organism at the level of individual cells [41].

A comprehensive investigation was conducted by Bensheng et al. to assess the suitability of zebrafish as a model organism for brain tumors. This was achieved by inducing the overexpression of a human variant of the oncogenic KRAS gene, specifically KRASG12V. By employing the promoters of zebrafish cytokeratin 5 (krt5) and glial fibrillary acidic protein (gfap) genes, they successfully induced the activation of Ras signaling within the CNS of zebrafish specimens. This was accomplished through the implementation of both transient and stable transgenic overexpression techniques. Immunohistochemical analyses were conducted to discern the activated pathways within the resultant brain tumors. The investigations demonstrated that zebrafish possess considerable potential as models for investigating the cellular underpinnings and molecular intricacies of brain tumorigenesis. Furthermore, zebrafish can serve as a valuable experimental system for comprehending the functional aspects of human oncogenes and for identifying novel inhibitors targeting the oncogenic RAS pathway [42].

The conventional methods employed for cancer modeling in zebrafish include genetic manipulation techniques as well as transplantation methodologies. Genetic manipulation methods can be further classified into reverse genetic strategies, such as genome editing or mutagenesis, as well as transgenesis. These methodologies enable the deliberate induction of gene-targeted mutations, thereby instigating a loss-of-function scenario for pivotal tumor suppressive genes. Alternatively, they facilitate the generation of a stable transgene that exhibits an augmented expression of an oncogene of particular interest. On the other hand, transplantation methodologies involve the judicious implantation of human cancer cells into an *in vivo* model organism, thereby providing a valuable experimental platform for further investigation. Zebrafish represent a highly advantageous alternative to traditional mouse models due to their enhanced efficiency and ease in conducting genetic modifications. Moreover, zebrafish enable the seamless execution of combinatorial functional investigations involving multiple genes, achieved through creating or merging various genetic variants. The utilization of zebrafish transgenic lines, which exhibit tissue-specific expression of a fluorescent protein, has been harnessed to gain additional elucidation regarding the domain of tumor biology. This encompasses the processes of tumor growth, and dissemination, the dynamic intricacies of tumor pathogenesis, and the molecular-level intricacies of the tumor microenvironment, all of which can be observed in real-time [43].

Aledst et al. have successfully devised a rapid zebrafish-centric PDX model, which is further enhanced by the implementation of longitudinal analysis using artificial intelligence (AI)-driven image processing techniques. This innovative approach effectively replicates crucial facets of glioblastoma growth and facilitates comparative evaluation of therapeutic agents. The researchers successfully performed the transplantation of 11 patient-derived GBM IDH wild-type cell cultures, which were genetically modified to express green fluorescent protein (GFP), into zebrafish embryos that were only one day old. The subsequent monitoring of the zebrafish was conducted using a state-of-the-art technique involving 96-well live microscopy and convolutional neural network analysis. Utilizing light-sheet imaging techniques to capture comprehensive data of whole embryos, the research team conducted an in-depth analysis of the invasive proliferation of neoplastic cells. The 11 primary tumor-derived cell lines (PDCs) exhibited notable heterogeneity in terms of growth, invasion, and survival characteristics. Furthermore, there was a strong correlation observed between tumor initiation in the PDCs and their corresponding PDX models in mice (Spearman's correlation coefficient, R = 0.89, p < 0.001). Zebrafish xenografts of GBM, under the surveillance of AI techniques within an automated framework, offer a scalable substitute to mouse xenograft models. This alternative enables the investigation of various aspects of glioblastoma tumor initiation, growth, and invasion, while also facilitating patient-specific drug evaluation [44]. Table 1 below summarizes some of the advantages and disadvantages of the *in vivo* models of brain tumors.

**Table 1:** Summary of *in vivo* models used to study brain tumors.

Model	Advantages	Disadvantages	Ref.
Rat C6 glioma model	- Emulates tumor growth and angiogenesis	- Limitations to faithfully reproduce the genetic intricacies - Causes rat mortality in 3–4 weeks	[35]
Orthotopic-xenograft GBM model	- Recapitulated the pathological and genomic characteristics of GBM - Identify distinct molecular signatures of GBM	- Limited physiological relevance, especially of the BBB	[36]
Endogenous model of mACVR1 glioma	- Faithfully reproduce GBM genotype - Faithfully reproduce GBM gene expression patterns	- Time consuming - High cost	[38]
Zebrafish Gal4-UAS model	- Excellent representation of the MAPK and PI3K pathways meticulous examination of temporal dynamics	- High cost	[40]
Zebrafish-PDX model incorporating AI image processing	- Effective emulate tumor angiogenesis and progression facilitating patient-specific drug evaluation - Facilitate patient-specific drug evaluation	- High computing power needed - Complex model to incorporate *in vivo.*	[42]

Table 2 provides a general comparison between rat, mouse, and zebrafish *in vivo* models for brain tumors.

**Table 2:** Comparison of *in vivo* models to study brain tumors.

Feature/Model	Rats	Mice	Zebrafish
**Advantages**	- Larger brain size for precise manipulations, Closely resemble human glioblastomas immunologically and transcriptionally	- Genetic manipulability, Cost-effectiveness, Availability of various transgenic strains	- Optical transparency in early development, Allows real-time visualization of tumor growth
**Suitability for Studies**	- Detailed anatomical observations, Studies requiring surgical precision	- Genetic studies, Recapitulating specific aspects of human brain tumors	- Tumor-induced vascular alterations, Drug delivery mechanisms
**Limitations**	- Higher costs, Greater ethical considerations	- May not replicate the complexity of human brain tumors	- Limited translational relevance to human conditions due to differences in mammalian brain tumor biology
**Instrumental Contributions**	- Enhancing the understanding of brain tumor biology and treatment responses	- Intracerebral tumor growth studies and understanding tumor progression	- Studying dynamic interactions between tumors and host tissues
**Comparative Costs**	- Higher due to size and maintenance	- Lower, more cost-effective	- Lower, but costs can vary based on specific experimental setups
**Ethical Considerations**	- Greater due to larger animal size	- Less compared to rats but still significant	- Lower, given the early life stage observation and smaller size
**Research Examples**	Brain Tumors in Man and Animals: Report of a Workshop (1986)	C57BL/6J mice used for studying malignant brain tumors (Wang et al., 2012)	Tumor-induced vascular alterations and drug delivery mechanisms in brain tumors (Doblas et al., 2010)
**Translational Relevance**	- High, due to anatomical and immunological similarities to human glioblastomas	- Moderate, benefits from genetic manipulability and diversity of transgenic models	- Moderate to Low, valuable for specific studies but limited by species differences in brain tumor biology

## *6* *In vitro* models of brain tumors

### 6.1 2-Dimensional models of brain tumors

2D cell models have proven crucial in brain tumor research, offering a simplified yet invaluable platform for investigating cancer development and progression. These models require the cultivation of tumor cells in a flat, two-dimensional layer, which enables convenient observation and manipulation of cells [9]. They are commonly used for drug evaluations and basic cancer research because they are simple and cost-effective. It is worth mentioning that 2D cell models do not possess the same level of complexity and physiological relevance as the *in vivo* tumor microenvironment [10]. This constraint can impact the precision of drug sensitivity assessments and the capacity to reproduce the behavior of tumor cells in a more realistic environment. In 2D cultures, tumor cells grow as a monolayer, which may not accurately capture the complex three-dimensional architecture and interactions found in real tumors [11]. Although these models have offered valuable insights into cancer biology, they may not fully capture the diverse characteristics and molecular patterns observed in brain tumors [12]. Inaccurately representing tumor characteristics can be a drawback due to the lack of spatial continuity in patch-wise designs [13]. Despite their limitations, 2D cell models are valuable for initial screenings and basic research in brain tumor studies. They provide a convenient starting point for exploring cancer cell behavior and responses to different treatments. Nevertheless, in order to acquire a more extensive grasp of tumor biology and drug responses, researchers are increasingly gravitating towards more sophisticated 3D models that closely resemble the *in vivo* tumor environment.

**Fig. 3:**
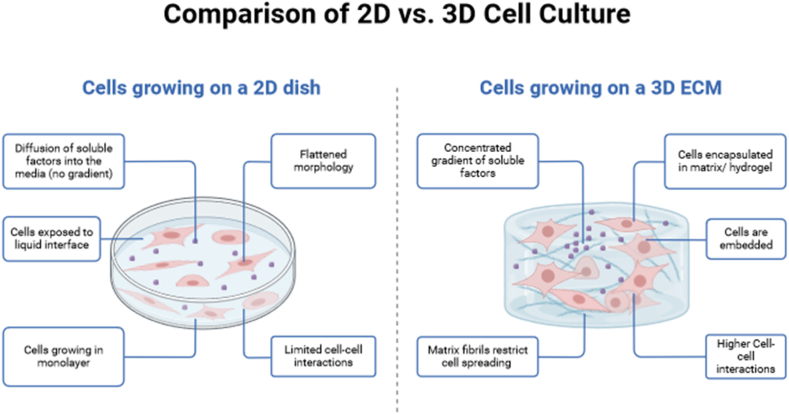
A generalized overview of the comparison between 2D and 3D cell culture.

The preclinical evaluation of drugs has predominantly relied upon the utilization of either 2D *in vitro* cellular models or animal-based studies. Nevertheless, 2D cultures suffer from certain limitations. These cultures, while convenient, do not faithfully replicate the complex *in vivo* tumor microenvironment [45]. Consequently, the reliability of such data is compromised. Furthermore, animal studies, despite their utility, are hindered by interspecies disparities that prevent a complete recapitulation of drug responses observed in humans. Creating 3D *in vitro* brain cancer models involves selecting relevant glioblastoma cell lines and cultivating them in conditions that mimic the extracellular matrix. Incorporating 3D scaffolds, microfluidic devices, and co-culture models with other cell types enables a more accurate representation of the *in vivo* tumor microenvironment. Bioreactors and perfusion systems support continuous nutrient supply, while advanced imaging techniques and functional assays monitor cell behavior. These models can be used for drug testing, biomarker analysis, and gaining insights into complex interactions within brain tumors. They offer a more physiologically relevant platform for studying brain cancer biology and testing therapeutic interventions compared to traditional 2D cell cultures. Fig. 3 gives a brief representation of 2D vs. 3D *in vitro* models of brain tumors.

### 6.2 3-Dimensional models of brain tumors

Therefore, 3D models that can simulate the brain tumor microenvironment more accurately are needed to fully replicate how the developed drug(s) work in humans. A study conducted by Gomez-Roman et al. devised a novel *in vitro* 3D-GBM model in which patient-derived glioblastoma stem cells (GSC) were cultured onto a polystyrene scaffold using Alvetex technology. In order to faithfully reproduce the perivascular stem cell niche, the cells were supplemented in a serum-free medium specifically formulated for stem cells, which was further enriched with vascular endothelial growth factor (VEGF), epidermal growth factor (EGF), and basic fibroblast growth factor (bFGF). The model was shown to emulate GBM in general, and the results of the model were comparable to GBM models derived from orthotopic xenografts [46]. Another model by Smith et al. used patient-derived GSCs to develop a 3D model of GBM tumor-mimetic hydrogel consisting of a Hyaluronic Acid (HA) backbone cross-linked with a collagen mimetic-matrix metalloprotease (MMP) degradable peptide, modified with cell-adhesive FN-mimetic peptides, and a physical gelling polymer, methylcellulose (MC). GSCs were seeded onto the hydrogel to study how GSCs migrated into the mimetic parenchymal invasion into the healthy tissues. The model demonstrated mechanisms identical to Extracellular Matrix (ECM) mediated chemotherapy and assisted in studying the downregulation of pro-apoptotic factors. Another study involved using 3D spheroids cultured in ultra-low attachment 96-well round-bottom plates. Spheroids were produced based on the UW228-3 medulloblastoma cell line, and the effectiveness was tested using 10 μM etoposide. The advantages of this model were that the results were highly reproducible, and it was possible to study tumor angiogenesis in the brain and crucial cellular processes in tumor progression [47].

In another study, SNB19 cellular entities were readily procured from the 3D construct assay and subsequently cultivated in both a two-dimensional monolayer configuration and neurospheres. Indeed, the SNB19 cells formed neurospheres after their initial cultivation within the three-dimensional model. The upregulation of CD133 and OCT4 was observed in the neurosphere and 3D assays, respectively. In contrast to cells cultured within the 2D model, cells exhibited heightened resistance to temozolomide within the 3D model, with this resistance being further augmented by the presence of hypoxia [48]. In a study by Jimenez et al., an *in vitro* model of GBM made from a U251-MG cell line was used to formulate a mathematical model to understand GBM tumor formation and progression. The cells were cultured in suspension for two weeks, after which the cell culture was infused with antibiotics, and the growth medium was loaded into a microfluidic chip to form a “biomimetic *in vitro*-in silico” model. Then, various equations pertaining to tumor cell growth and proliferation were used to model GBM progression, and the results from the model were compared to *in vitro* results from the microfluidic chip. The study reported that the model was able, to a large extent, to extract the fundamental parameters that lead to the GBM progression. However, there were a few discrepancies, such as a lag between the experimental and computational results, which were attributed to the lag time for the *in vitro* cells to proliferate and mutate from their original form [49].

Various glioblastoma 3D *in vitro* cell culture models have been devised to recapitulate the tumor microenvironment and emulate the interactions between tumor cells, diverse cellular constituents, and the extracellular matrix. A study by Lee et al. showed that *in vitro* models comprising Tumor Stem Cells (TSCs) derived directly from primary glioblastomas exhibit a remarkable resemblance to normal neural stem cells. Moreover, these TSCs faithfully reproduce human glioblastomas' genotype, gene expression patterns, and *in vivo* biology. Tumor cells cultivated under conventional serum-based cell culture conditions exhibit a depletion of and consequently give rise to a cohort of cells that exhibit substantial genetic and biological disparities compared to the original tumors from which they originated. The results of this study indicate that TSCs exhibit more significant potential as a model system compared to frequently employed cancer cell lines in comprehending the intricacies of primary human tumors [50]. A team led by Jeremy Rich et al. successfully developed three-dimensional organoids derived from human GBM cells and GBM biopsies. Upon embedding finely minced GBM specimens within PGMA matrigel, these specimens exhibited a growth of up to 3–4 mm within a span of two months. Remarkably, these organoids could be maintained in culture for long durations. However, it is worth noting that their growth rate gradually diminished after several months, which was attributed to the restricted diffusion of essential nutrients as the organoids expanded in size [51]. An intriguing investigation conducted by Linkous et al. has successfully cultivated patient-derived glioblastoma cells within cerebral organoids and multicellular constructs that faithfully replicate the architectural intricacies of a developing human brain. The nomenclature assigned to this particular model is GLICO, an acronym derived from cerebral organoid glioma. The glioblastoma cells utilized for generating GLICOs were cultured from tumor tissue obtained from patients, employing specific conditions devoid of serum, which facilitate the sustenance of a stem cell-like characteristic and are commonly denoted as GSCs. As previously elucidated, the cerebral organoids were cultivated utilizing human embryonic or induced pluripotent stem (iPS) cells, employing methodologies that facilitate three-dimensional amplification of neuroectoderm. When glioblastoma stem cells (GSCs) were subjected to co-cultivation with fully developed cerebral organoids, it was observed that the GSCs exhibited a remarkable propensity for invasion and successfully established tumors within the organoids. This phenomenon was observed with striking efficiency, as evidenced by a 100 % tumor take rate. These findings underscore the inherent invasive characteristics of glioblastoma cells and highlight the ability of GSC conditions to maintain and preserve this invasive capacity during cell culture [52].

In another study, Choe et al. developed a model that aimed to simulate brain tumor metastasis. The investigation successfully devised an uncomplicated 3D *in vitro* model to accurately mimic brain metastasis. This was accomplished by using human cancer cells in conjunction with cerebral organoids derived from human embryonic stem cells, thereby yielding what is referred to as metastatic brain cancer cerebral organoids (MBCCO). The MBCCO model effectively replicated the dynamics of metastatic brain cancer, encompassing crucial phenomena such as cellular adhesion, proliferation, migration, and intercellular interactions [53]. Fan et al. proposed an innovative 3D-GBM cell culture model utilizing microwells to replicate the *in vitro* environment. Microwells were fabricated using a cost-effective and readily available polyethylene glycol (PEG) material, thereby enabling precise manipulation and regulation of *in vitro* 3D culture systems. The 3D micropatterning system was employed to manipulate GBM (U-87) cells through the utilization of the photolithography technique, thereby exerting precise control over the morphological characteristics of the cell spheroids, including their shape, size, and thickness. Based on their initial findings, it was observed that the formation of homogeneous GBM spheroids can be achieved in a 3D environment. Furthermore, it was determined that the dimensions of these GBM spheroids are contingent upon the size of the microwells utilized in the experimental setup. The quantitative assessment of the spheroids' viability, generated through the methodology above, was conducted using a live/dead assay and demonstrated a progressive enhancement in viability over a period of 21 days [54]. A subsequent investigation conducted by Lakkadwala et al. entailed the development of an *in vitro* model for brain tumors, wherein a biodegradable scaffold composed of PLGA-chitosan was employed. A 3D glioblastoma tumor was cultivated within the scaffold, and afterward, the evaluation of liposome transport through the brain endothelial barrier into the 3D tumor was conducted. The primary benefit of this particular model lies in its ability to faithfully replicate *in vivo* conditions, thereby simulating the scenario where the delivery carrier, following systemic administration, must initially traverse the BBB prior to ultimately reaching the intended tumor sites. Furthermore, it is worth noting that the co-culture brain endothelial model, consisting of brain endothelial and glial cells, exhibited a remarkable capacity to establish a highly impermeable barrier. This phenomenon can be attributed to the enhanced expression and functionality of junctional proteins, which were facilitated by the presence of glial cells. Consequently, the physical integrity and robustness of the barrier were significantly bolstered. Utilizing the present model, the researchers assessed the effectiveness of liposomal nanoparticles that possess dual functionalization in traversing the co-culture brain endothelial barrier. Furthermore, these nanoparticles were investigated for their ability to deliver 5-FU in a highly efficient manner to the U87 tumor cells residing within the PLGA-chitosan scaffold, ultimately leading to the eradication of the tumor cells [55]. Meng et al. established a 3D-brain tumor co-culture model using Poly (Glycerol-Adipate) nanoparticles. This model comprised brain tumor aggregates made up of DAOY cells and organotypic brain slices. These aggregates showed adhesion to cerebellum slices and penetrated as a unified entity. Cellular entities then disengaged from the aggregates, effectively replacing native brain cells. The dissection and subsequent culture of cerebellum slices had been performed using DAOY monolayer cells and magnetic microspheres. These slices were coated with DAOY aggregates ranging in size from 200 to 300 μm [56].

In another investigation, Dai et al. successfully devised an innovative experimental approach involving the development of a 3D bioprinted model for glioma stem cells. This model was constructed utilizing a meticulously engineered porous hydrogel composed of gelatin, alginate, and fibrinogen, which was specifically modified to closely resemble the structure and composition of the extracellular matrix. The glioma stem cells exhibited a remarkable survival rate of 86.92 %, demonstrating their robustness during bioprinting procedures. Moreover, these cells displayed a notable increase in cellular activity, indicating their propensity for rapid proliferation upon being subjected to the bioprinting process. Throughout the *in vitro* cultivation phase, it was observed that the glioma stem cells that were subjected to printing techniques exhibited a remarkable ability to retain their intrinsic properties as cancer stem cells, as evidenced by the sustained expression of Nestin. Furthermore, these printed glioma stem cells also demonstrated a noteworthy capacity for differentiation, as indicated by the expression of glial fibrillary acidic protein and β-tubulin III, which are markers associated with glial and neuronal differentiation, respectively. To ascertain the vascularization potential of glioma stem cells, the immunohistochemical method was employed to detect the presence of vascular endothelial growth factor (VEGF), a biomarker associated with tumor angiogenesis. It was observed that the expression of VEGF exhibited a progressive increase from the first week to the third week of the culture period. The findings of the drug-sensitivity analysis revealed that the tumor model created through 3D printing exhibited a higher level of resistance towards temozolomide when compared to the 2D monolayer model. This disparity in sensitivity was explicitly observed at concentrations of temozolomide ranging from 400 to 1600 μg/mL In brief, the utilization of a 3D bioprinted glioma model showed a promising and innovative approach for investigating various aspects of gliomagenesis, glioma stem cell biology, drug resistance mechanisms, and the efficacy of anticancer drugs in an *in vitro* setting [57].

A study by Tricini et al. presented a novel 1:1 scale 3D-printed biohybrid model that faithfully replicates the microenvironment of brain tumors. This model encompasses both the luminal and parenchyma compartments, providing a comprehensive representation of the tumor's complex architecture utilizing a microfluidic device that exhibits dynamic control capabilities, achieved through the utilization of a two-photon lithography fabrication technique. This facilitates the simultaneous co-culture of three distinct cell types, namely hCMEC/D3 cells, which compose the internal biohybrid endothelium of the capillaries, astrocytes, and magnetically-driven spheroids of U87 glioblastoma cells. Tumor spheroids were procured by cultivating glioblastoma cells within three-dimensional microcages laden with superparamagnetic iron oxide nanoparticles (SPIONs). The experimental findings demonstrated the system's efficacy in impeding the diffusion of dextran molecules across the bioinspired BBB, while simultaneously facilitating the passage of nanocarriers loaded with chemotherapy agents [58]. Table 3 below summarizes some of the advantages and disadvantages of the *in vitro* models of brain tumors.

**Table 3:** Summary of *in vitro* models used to study brain tumors.

Model	Cell Type	Scaffold	Application	Advantages	Disadvantages	Ref
3D-GBM model on a polystyrene scaffold	U 87 – GBM cells	Polystyrene scaffold (Alvetex)	Drug study and Disease Modelling	- Emulates GBM in general - Similar to orthotopic xenograft - Controlled tumor environment	Simplification of complexity	[21]
3D-GBM model using HA crosslinked with MMP	Patient-derived glioblastoma stem cells (GSC)	1%w/v HA-methylfuran hydrogel	Disease modeling	- Mimics parenchymal invasion into healthy tissues - Showed ECM-mediated chemotherapy effects	Limited physiological relevance	[22]
3D- TSC model	Human- derived tumor stem cells	Not provided	Disease modeling	- Faithfully reproduce GBM genotype - Faithfully reproduce GBM gene expression patterns - Cost-effective	Absence of a dynamic BBB	[24]
GBM Matrigel-organoid	Human Pluripotent Stem Cells (Hpsc)	Matrigel	Disease modeling	- Organoids maintained in culture for a long time (3 months) - Easily reproducible	Growth rate diminishes after several months	[25]
3D-GLICO	Patient-derived glioblastoma stem cells (GSCs)	Matrigel	Disease modeling	- Shows remarkable tumor invasion 100 % tumor uptake rate	Static nature Potential for artifacts	[26]
3D-MBCCO	Human embryonic stem cell-derived cerebral organoids	Not applicable	Drug Screening	- Effectively replicated the dynamics of metastatic brain cancer - Excellent cellular adhesion, proliferation, migration	Highly dependent on the cell line used	[29]
PLGA-chitosan scaffold GBM model	U87 tumor cells	PLGA chitosan scaffold	Drug screening	Faithfully replicate *in vivo* conditions Effective BBB modeling	No dynamic blood supply	[30]

## 7 Microfluidic models of brain tumors

Microfluidics involves manipulating and controlling fluids within small channels, typically ranging from microliters to picoliters in volume and tens to hundreds of micrometers in size. This technology allows for the precise manipulation and examination of minuscule fluid volumes, proving extremely valuable in a wide range of disciplines such as chemistry, biology, diagnostics, and pharmaceuticals. Its applications include lab-on-a-chip devices, point-of-care testing, and single-cell analysis. Research into brain tumors, and more specifically, the modeling of brain tumors for different purposes, has greatly benefited from microfluidics. Researchers have been able to recreate the complex physiology of solid tumors in a controlled environment thanks to the development of microfluidic systems, such as tumor-on-chip devices. These microfluidic platforms enable the creation of multicellular tumor spheroids for drug testing, offering a more physiologically relevant model for assessing anti-cancer treatments. In addition, researchers have used microfluidic organ-on-a-chip systems to evaluate the effectiveness of chemotherapy in brain tumors.

Photolithography for microfluidic fabrication involves coating a substrate with a light-sensitive photoresist and then exposing it to UV light through a patterned mask. Exposed areas of the photoresist are developed away, revealing the substrate beneath. This exposed substrate is then etched to create microchannels, and the remaining photoresist is removed, resulting in a microfluidic device with precise patterns. Microfluidic devices may be fabricated via soft lithography, which entails the production of molds from an elastomer, usually polydimethylsiloxane (PDMS), using a master design. The PDMS is applied onto the master material, subjected to curing, and then removed to expose microscale channels and structures. This approach is adaptable, economical, and well-suited for quick prototyping. 3D-printed microfluidic chips are manufactured using a process of layer-by-layer printing, enabling the creation of [59] three-dimensional structures without the use of masks or molds. This method facilitates rapid design refining and personalization, but requires meticulous choice of printing materials to ensure compatibility with particular applications.

**Fig. 4:**
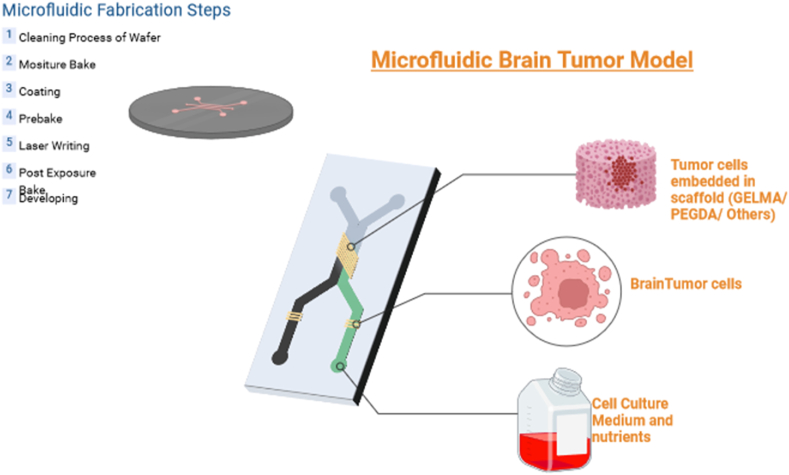
A generalized overview of the workings of a microfluidic-based brain tumor model.

In recent years, there has been a surge in the use of microfluidic models in brain tumor research. These models have proven to be highly influential due to their ability to provide a sophisticated platform that faithfully replicates the complex microenvironment of the central nervous system. The utilization of microscale systems enables researchers to replicate the physiological and biochemical milieu present in the brain, thereby facilitating a more precise emulation of tumor development and progression [60]. Through the amalgamation of microfluidic platforms with 3D cell cultures, scholars can replicate the diverse nature of cerebral neoplasms, thereby facilitating an all-encompassing exploration of the dynamic interplay among neoplastic cells, adjacent tissues, and the vascular system. The meticulous regulation of fluid dynamics and the capacity to introduce gradients of signaling molecules serve to augment the accuracy of these models, thereby enabling a nuanced investigation into the processes of tumor cell migration, invasion, and response to therapeutic interventions [61]. Microfluidic platforms have shown great promise in improving our basic understanding of brain tumors and in speeding up the development of effective and highly specific targeted treatments [62]. Fig. 4 gives a generalized overview of a microfluidic chip that aims to emulate brain tumors.

### 7.1 Models fabricated using soft-lithography

Truong et al. developed a microfluidic model to study GBM vasculature, using CAD software and a transparent mask. The microfluidic chip was fabricated using soft-lithography on PDMS. The model consists of three concentric semicircles with trapezoidal micro-posts for inter-regional interactions. The device's microvascular network influences the migration of glioblastoma stem cells within a hydrogel matrix, promoting invasive cellular morphology while preserving proliferation rates. The study also analyzed migration behavior in an *in vivo* mouse model [63]. The microfluidic chip developed by Han et al. comprised a series of 488 hexagonal chambers interconnected by two microchannels. This design facilitates the efficient and convenient long-term cultivation of cells, specifically targeting drug-resistant GBM cells. The chip was fabricated using soft lithography techniques with PDMS. The chip allowed for the daily replenishment of doxorubicin (DOX) and culture media, enabling researchers to gain insights into the underlying mechanisms of drug resistance in GBM cells. The U87 cells, uniformly dispersed within the chambers, exhibited a notable reduction in cell count in close proximity to the DOX channel on the third day following treatment. Subsequently, three-fourths of the chambers were devoid of cells by the fifth day. However, cell repopulation was observed by the seventh day, suggesting the emergence of resistance and the migration of resistant cells towards regions with elevated DOX concentration [64]. In a study conducted by Mamani et al., magnetic hyperthermia therapy (MHT) was employed to administer localized heat to treat cancerous cells. The study employed a microfluidic device in conjunction with a chip platform to cultivate GBM tumors, thereby providing a controlled and representative experimental environment. Utilizing the modality of MHT, tumor cells engaged in interactions with magnetic nanoparticles (MNP) were subjected to the influence of an oscillating magnetic field. Subsequently, an exhaustive examination of the cellular viability within the temperature range of 41 °C–43 °C was conducted. The innovative design facilitates the occurrence of cell-cell interactions and enables the cultivation of cells in a 3D environment. The utilization of microchannels facilitated the propagation of fluidic motion within the culture medium, thereby emulating the dynamics of the tumor microenvironment *in vivo*. This was achieved by precisely manipulating magnetic nanoparticles, which were strategically directed toward cancerous cells for targeted therapy. In contrast to conventional 2D cell cultures, it was observed that cell cultures cultivated within a suitable cavity in the chip exhibit a remarkable degree of similarity to *in vivo* tumor cells [65]. Fuchs et al. devised a microfluidic model for simulating interactions between neurons and pHGG cells. The chip was fabricated using soft lithography techniques with PDMS. They achieved this by co-culturing human-induced Pluripotent Stem (hiPS)-derived cortical glutamatergic neurons and pHGG cells in compartmentalized microfluidic devices. Additionally, they developed a method to record the electrophysiological changes in these cells. The model successfully distinguished and classified human glutamatergic neurons. Subsequently, the model included the brain microenvironment and neuronal activity to examine the electrical influence of pHGG cells on these neurons inside the milieu.

### 7.2 Models fabricated using photo-lithography

The groundbreaking work by Fan et al. yielded a pioneering 3D brain cancer chip, that showcased four reservoirs, comprising three inlets and one outlet. This chip design was fabricated using photo-lithography by a PEGDA hydrogel layer, strategically positioned between the top and bottom cover glasses. The hydrogel exhibited permeability to a diverse range of molecules, including water, biomolecules, and chemicals. Furthermore, it had the ability to regulate the release of chemicals through diffusion. This facilitated the formation of GBM spheroids in a high-throughput manner, the administration of multiple drugs simultaneously, and the conduction of extensive parallel assessments of drug responses. In the study, GBM cells were successfully cultivated on the microfluidic chip platform. Simultaneous administration of two commonly employed chemotherapeutic agents, namely Pitavastatin and Irinotecan, was performed within the hydrogel matrix through the utilization of two distinct inlets. The drug exhibited a consistent release profile from the hydrogel matrix until the concentration within the micro-well reached a steady state, remaining unaffected by the periodic replacement of drug-free media. The assessment of drug efficacy, as quantified by the cellular viability of the spheroids, revealed that the combined administration of both drugs exhibited significantly superior effectiveness in comparison to the individual drug treatments [66]. [64,65].

Lee et al. employed a droplet-based microfluidic platform to fabricate 3D tumor spheroids, with diameters ranging from 100 to 130 μm. These spheroids were specifically composed of U87 GBM cells and were utilized for the purpose of conducting photothermal therapy. The control of spheroid size can be achieved through two distinct approaches: the manipulation of droplet volumes contingent upon the flow rate in the junction, or the modification of cell concentration. The microfluidic chip successfully demonstrated its capability to generate a substantial number of droplets, amounting to an impressive count of 42,000. This generation yield surpassed that of comparable setups by approximately 20 %, highlighting the chip's superior performance. The droplet generation process was accomplished within a time frame of 10 min, showcasing the chip's efficiency. Notably, a remarkable 80 % of the generated droplets encapsulated spheroids, indicating the chip's proficiency in producing desired structures. It is important to note that these results were achieved under optimal conditions, specifically when the oil flow rate was set to its maximum value of 50 μL/min [67]. Lee et al. successfully devised a sophisticated tumor-on-a-chip model utilizing GelMA hydrogel as the substrate for co-culturing GBM (U87MG) and breast (MCF7) cancer cells. This innovative approach enables the investigation of cancer metastasis dynamics and the assessment of photothermal therapy (PTT) effectiveness. The investigation encompassed the execution of both pressure transient testing (PTT) and migration studies within a unified microfluidic platform, which was meticulously fabricated through a two-step photolithography process. Following the application of NIR laser irradiation, it was observed that the cellular viability of MCF7 and U87MG cells, which had been subjected to a 20 v/v% concentration of gold nanorods, experienced a substantial reduction from approximately 90 % to less than 10 %. This decrease in viability was consistent across both cancer cell types [68]. Truong et al. have devised a cutting-edge 3D organotypic microfluidic platform, which was integrated with hydrogel-based biomaterials. This sophisticated platform has been ingeniously designed to faithfully replicate the characteristics of the glioblastoma stem cell (GSC) vascular niche. The primary objective of this study was to comprehensively investigate the impact of endothelial cells (ECs) on the behavior of GSCs derived from patients. The researchers were able to observe the precise signaling cues that orchestrate the invasive nature and phenotype of these GSCs. The augmentation of the pre-existing microvascular network facilitated the migration of GSCs within a 3D hydrogel matrix. Additionally, this phenomenon promoted the adoption of an invasive cellular morphology by the GSCs, while simultaneously preserving their rates of proliferation and phenotypic characteristics [69]. Another study by Sowah et al. focused on creating a microfluidic model called organotypic triculture, which aimed to replicate the perivascular niche (PVN) and investigate its impact on GSCs. The triculture system, consisting of ECs, astrocytes, and GSCs, was used to examine the invasion, proliferation, and stemness of GSCs. The coexistence of both ECs and astrocytes greatly enhanced the invasiveness of GSCs. In addition, this work used single-cell RNA sequencing (scRNAseq) to identify 15 ligand-receptor pairings that are linked to potential chemotactic processes of GSCs. Within these pathways, it was shown that the receptor was increased in expression in GSCs, while the diffusible ligand was discovered to be expressed in either astrocytes or ECs [70].

In another study, Amemiya et al. used the capabilities of microfluidic systems to construct a comprehensive and authentic model of vasculogenesis in GBM. Two PDMS devices were fabricated, namely a doughnut-hole dish and a 5-lane microfluidic device, for the purpose of investigating the contact-independent effects of GBM cells on human umbilical vein endothelial cells (HUVECs). A total of ten cell lines were utilized in this study, consisting of five patient-derived GBM cell lines and five widely employed GBM cell lines. This study was able to elucidate novel insights into the neovasculogenic mechanism of GBM [71].

Silvani and his team created a 3D-bioprinted model of GBM-on a chip, replicating the pathophysiological conditions of a brain tumor. The model was created using a microfluidic network and photolithography mold on PDMS. It had three compartments, each serving a specific purpose. The central compartment was for tissue cultivation and observation, while the vascular channel facilitated nutrient and waste exchange. The device also had two microfluidic channels for fluid flow control. The model was designed for applications like media perfusion, drug testing, and incorporating diverse cell types like astrocytes. The GBM-on-a-chip successfully recreated the complex interplay of biochemical and mechanical stimuli [72]. Table 4 provides a holistic comparison between *in vitro*, *in vivo* and microfluidic tumor models for brain tumors.

**Table 4:** Summary of the microfluidic models used to study brain tumors.

Model	Cell Type	Scaffold	Application	Advantages	Disadvantages	Ref
3D-PDMS GBM on a chip	Human Glioblastoma cells A-172	Gelatin methacryloyl (GelMA)-Alginate	Disease Modelling	- Controlled microenvironment - Replicates the microenvironment of the central nervous system	- Simplification of *in vivo* complexity - Absence of certain physiological elements of the real tumor microenvironment - Fabrication is difficult	[56]
3D-PEGDA hydrogel chip	U 87 GBM cells	PEGDA Hydrogel	Disease Modelling	- Mimics tumor heterogenicity - High throughput drug screening	- Careful control of the polymerization process needed for the formation of PEGDA hydrogel - Device clogging and fouling affect reliability	[57]
Dual Channel GBM on a chip	Human Vascular Endothelial Cells, Brain Pericytes	BisSR 3D polymer scaffold	BBB modeling	- Excellent modeling of brain tumor dynamics - Able to recapitulate mechanical and biological stimuli	- Requires expensive material and time-consuming fabrication process - The device is quite complex, with multiple channels that make fine control difficult	[58]
GELMA-Hydrogel GBM on a chip	U 87 GBM cells	Gelatin methacrylate (GELMA) hydrogel	Disease modeling	- Substantial reduction in tumor size - Promoted the adoption of an invasive cellular morphology by the GSCs, while simultaneously preserving their rates of proliferation and phenotypic characteristics.	- Oversimplification of tumor complexity - Time-consuming to fabricate - Expensive	[59]
3d Bioprinted GBM model	Glioblastoma Stem Cells (GSC)	Not provided	Disease Modelling			[60]
Microfluidic droplet-based GBM spheroids	U 87 GBM cells	Not provided	Disease Modelling	- Precise control and manipulation of fluid flow	- GBM spheroids need to be produced outside the microfluidic systems - Sometimes compartmentalization affects the nutrients available for the spheroids to grow, which can change the tumor morphology	[61]

Table 5 summarizes a holisitic comparison between *in vitro*, *in vivo* and microfluidic tumor models for brain tumors.

**Table 5:** Comparison of *in vitro*, *in vivo* and microfluidic tumor models for brain tumors.

Aspect	*In vitro* Models	*In vivo* Models	Microfluidic Models
**Setting/Environment**	Laboratory setting outside a living organism	Within a living organism	Microscale technologies mimicking the tumor microenvironment
**Primary Strengths**	- Cost-effective, - Suitable for high throughput screening, - Precise control over variables	- Comprehensive view of tumor behavior, - Can assess the effectiveness of therapies, - Reflects tumor-host interactions	- Combines advantages of *in vitro* and *in vivo*, - Precise control over experimental conditions, - Can simulate physiological contexts more accurately
**Primary Limitations**	- May not replicate the complexity of *in vivo* systems, - Lack of physiological context	- High cost,- Ethical considerations,- Time-consuming	- While advanced, may still not capture all aspects of *in vivo* environments, - Technological and accessibility barriers for some research settings
**Applications**	- Initial drug screening, - Mechanistic studies	- Effectiveness of potential therapies, - Understanding tumor-host interactions	- Enhancing comprehension of tumor biology and drug responses, - Studying tumor behavior in physiologically relevant settings
**Physiological Relevance**	Low; lacks the complexity and interactions present in a living organism	High; provides a holistic view of the biological and chemical interactions within the living organism	Moderate to High; designed to closely replicate key aspects of the tumor microenvironment
**Resource Intensity**	Low to Moderate; cost-effective and requires less specialized equipment	High; requires significant resources for animal care, ethical approvals, and specialized equipment	Moderate; requires specialized microfabrication and analysis equipment but can be cost-effective compared to *in vivo* studies
**Ethical Considerations**	Minimal; does not involve live animals	Significant; involves the use of live animals and associated ethical considerations.	Minimal to None; reduces reliance on animal models
**Throughput Capability**	High; allows for the screening of large numbers of compounds or genetic conditions in a shorter time frame.	Low to Moderate; limited by the number of animals that can be feasibly and ethically used	Moderate to High; can process multiple samples simultaneously, though typically less than traditional *in vitro* methods.
**Complexity of Tumor Microenvironment Simulation**	Low; primarily focused on cellular behavior without replicating the full microenvironment	High; naturally includes the complex interactions within the microenvironment.	High; specifically designed to mimic the tumor microenvironment, though still an approximation
**Future Directions**	Incorporation of advanced biomaterials. Patient derived cells, and organoids	Development of genetically engineered models, patient-derived xenografts (PDX)	Incorporation of patient-derived cells, high throughput screening

## 8 Artificial intelligence (AI) models for brain tumors

The advent of machine learning (ML) and AI has revolutionized a new revolution in the modeling of the brain tumor microenvironment. ML provides a multitude of benefits, including the ability to seamlessly integrate data from various sources, enabling a comprehensive understanding of the intricacies involved in tumor progression. These models demonstrate exceptional proficiency in accurately forecasting tumor behavior and treatment outcomes, streamlining the process of image segmentation through automation, and effectively extracting pertinent features from datasets [73].

The inclusion of AI into brain tumor models has greatly propelled the field of neuro-oncology. AI technologies are being used increasingly to improve precision, effectiveness, and customized approaches to diagnosing brain tumors, planning treatment, and predicting prognosis. Through the use of AI algorithms, researchers and clinicians have the ability to analyze medical imaging data, including MRI scans, in order to accurately detect, classify, and segment brain tumors [74]. AI models utilizing deep learning techniques have made remarkable advancements in automated medical image diagnosis. This has resulted in faster and more precise identification of brain tumors from imaging data [75]. In addition, AI has been instrumental in advancing radiomic and radiogenomic analyses for brain tumors. Through the analysis of medical images and the incorporation of genomic data, AI algorithms have the potential to offer valuable insights into the molecular makeup of brain tumors. This information can greatly assist in the process of tumor characterization and treatment planning [76].

AI has been instrumental in advancing brain tumor modeling, allowing for the real-time monitoring of tumorigenesis and the analysis of the tumor microenvironment. Advanced systems, combined with AI algorithms, enable the thorough evaluation of biophysical tumor properties and the evolving patterns in tumor growth, opening up possibilities for personalized medicine strategies in brain tumor management, as well as the data obtained from such analysis can be used to model brain tumors [77].

Ultimately, the incorporation of AI into brain tumor models has made remarkable strides in the realm of neuro-oncology, bolstering the precision of diagnoses, treatment strategies, and personalized medicine approaches. Through the utilization of AI technologies, researchers and clinicians have the ability to extract valuable insights from medical imaging data for brain tumor modeling.

## 9 Discussion and concluding remarks

Brain tumors are some of the most formidable cancers originating in the CNS among the human population, lacking efficacious therapeutic interventions or definitive remedies. Despite five decades of extensive research and meticulous observations, the scientific community has regrettably failed to achieve any noteworthy breakthroughs in terms of enhancing patient survival rates subsequent to the diagnosis of GBM. The robust cellular infiltration observed in brain tumors has thus far proven to be a formidable challenge for both surgical and therapeutic interventions. Despite significant efforts, these interventions have been unable to completely eradicate tumor cells, resulting in residual cells within the brain tissue that subsequently give rise to new neoplasms within a relatively short timeframe following surgical resection [78]. The efficacy of investigational drug studies is frequently compromised by the inherent challenges posed by cellular and genetic heterogeneity, which can impede the desired therapeutic outcomes. Additionally, the presence of the BBB further restricts the delivery of drugs to the specific affected structures, exacerbating the limitations already imposed on treatment strategies. While histopathological analyses remain the primary method for diagnosing GBM, the utilization of patient-derived tumor tissues has revealed substantial cellular, genomic, proteomic, and extracellular heterogeneity. Consequently, the analysis of such heterogeneity necessitates the implementation of multiscale analysis methods. Numerous *in vitro*, *in vivo* and microfluidic models have been developed to better understand the brain tumor microenvironment, and develop innovative strategies that can mitigate the devastating consequences of this disease.

The utilization of *in vitro* models for the study of brain tumors presents many advantageous attributes, encompassing the provision of a meticulously regulated environment with precise experimental parameters and commendable cost-effectiveness when juxtaposed with *in vivo* models. The circumvention of ethical considerations pertaining to animal testing contributes to the broader accessibility of this practice. The utilization of these models confers notable benefits in the context of high-throughput screening of putative therapeutic agents, thereby expediting the replication of findings and allowing investigations at the molecular and cellular levels. *In vitro* models, in addition to their inherent advantages, can isolate and investigate distinct cellular subpopulations implicated in cerebral neoplasms, facilitating facile genetic manipulation and dynamic visualization of intracellular events. Nevertheless, these aforementioned advantages are accompanied by inherent limitations. *In vitro* models frequently exhibit a reductionist approach by simplifying the nature of the *in vivo* tumor microenvironment, thereby failing to encompass the intact tissue architecture and complete physiological significance inherent in *in vivo* conditions. The utilization of artificial culture conditions, the lack of interactions with immune cells, and the inherent difficulty in reproducing dynamic changes within a static system are prominent drawbacks that warrant attention. Moreover, it is imperative to acknowledge that translating discoveries from *in vitro* models to practical clinical applications is not without its inherent challenges. Furthermore, the task of faithfully reproducing the complete spectrum of heterogeneity observed in brain tumors within an *in vivo* setting continues to present a noteworthy limitation [[79], [80], [81]]. Notwithstanding these limitations, *in vitro* models serve as invaluable instruments in the investigation of cerebral neoplasms, augmenting and enlightening more extensive research endeavors. Subsequent advancements may involve the integration of sophisticated biomaterials that more closely resemble the microenvironment of brain tissue into 2D cell culture models representing brain tumors. Furthermore, the utilization of patient-derived cells in 2D monolayers could facilitate personalized drug screening and provide valuable insights into the unique responses of individual tumors. Advancements in 3D cell culture may include the creation of intricate models including several cell types to more accurately replicate the tumor microenvironment. Additionally, the use of organoid technology for customized medicine and drug testing may be pursued. In order to investigate tumor angiogenesis and its interactions with the BBB, it is possible to replicate vascularization in 3D culture models.

The utilization of *in vivo* models to study brain tumors offers a highly sophisticated and platform that effectively captures the realistic features inherent to the microenvironment of the human brain. The utilization of these sophisticated models provides a high degree of physiological relevance, thereby facilitating the investigation of metastatic processes, tumor progression dynamics, and the behavioral aspects associated with brain tumors [82]. Consequently, this heightened physiological fidelity significantly enhances the translational potential of these models, thereby bolstering their applicability in clinical settings [79]. Nevertheless, the utilization of *in vivo* models poses considerable challenges, encompassing exorbitant expenditures linked to the maintenance of animals, ethical considerations, and a labor-intensive character. The utilization of *in vivo* models for brain tumor research necessitates meticulous deliberation due to several factors. These include the presence of limited experimental control, variations between species, difficulties associated with genetic manipulation, and the inherent variability observed among animals [83,84]. The development of genetically modified mice models that more accurately replicate human tumors, together with the growing use of patient-derived xenografts for clinically relevant drug testing, might enhance the effectiveness of *in vivo* models of brain cancers. Recent developments in imaging techniques, like as multiphoton microscopy, have the potential to provide enhanced resolution for seeing tumor growth and assessing therapy efficacy in live animal models.

Microfluidic models of brain tumors provide a carefully controlled and physiologically important platform, effectively emulating the microenvironment of the central nervous system. The utilization of precise experimental conditions offers a multitude of advantages, encompassing the ability to exert meticulous control over various parameters. This level of control facilitates the replication of tumor heterogeneity, thereby enabling researchers to simulate the complexities inherent to tumors. Moreover, this approach holds immense potential for conducting high-throughput drug screening, thereby expediting the process of identifying efficacious therapeutic agents [85]. The integration of organ-on-a-chip platforms into microfluidic models has the potential to boost their skills in simulating the interactions between the blood-brain barrier and tumor-vascular systems. Additionally, the incorporation of high-throughput screening capabilities may facilitate the quick evaluation of therapeutic effectiveness and toxicity in brain tumor models.

Nevertheless, one must acknowledge the inherent challenges that arise in the pursuit of studying complex biological phenomena *in vivo*. These challenges primarily revolve around the necessity to simplify the intricacies inherent *in vivo* systems, the unavoidable absence of certain physiological elements that are present *in vivo*, and the imperative requirement for meticulous validation procedures to ascertain the relevance and applicability of *in vitro* findings to the *in vivo* environment [86]. It is imperative to acknowledge that these models make a significant contribution by providing invaluable insights into the dynamics of brain tumors and their corresponding therapeutic responses within a meticulously controlled and reproducible experimental framework [87].

Furthermore, for ML and AI to be widely accepted as an integral component of tumor modeling, a crucial prerequisite is the rigorous validation through meticulous clinical assessments. Numerous studies have been published in scientific literature involving the use of ML models to accurately classify and predict brain tumors. Although using AI and ML models to accurately depict the brain tumor microenvironment is still in its infancy, with the development of novel technologies, such as digital twins, it is highly likely that we can obtain a much more accurate model of brain tumors, which could potentially translate to more effective treatment regimens and an improved prognosis of this dismal disease.

## Data availability

This is a review article, so no data was taken.

## CRediT authorship contribution statement

**Richu Raju R:** Writing – review & editing, Writing – original draft, Methodology, Conceptualization. **Nour M. Al Sawaftah:** Writing – review & editing, Supervision, Resources, Project administration, Methodology, Funding acquisition, Conceptualization. **Ghaleb A. Husseini:** Writing – review & editing, Conceptualization.

## Declaration of competing interest

The authors declare that they have no known competing financial interests or personal relationships that could have appeared to influence the work reported in this paper.
